# Effects of Future Subjective Expectations on Cognitive Decline and Dementia among Middle-Aged and Older Adults

**DOI:** 10.3390/bs14050421

**Published:** 2024-05-17

**Authors:** Minsung Sohn, Xianhua Che, Hee-Jung Park

**Affiliations:** 1 Division of Health and Medical Sciences, The Cyber University of Korea, Seoul 02708, Republic of Korea; minsinge@cuk.edu; 2 Department of Health Policy Research, Daejeon Public Health Policy Institute, Daejeon 35015, Chungcheong-do, Republic of Korea; che719@djpi.or.kr; 3 Department of Dental Hygiene, College of Health Science, Kangwon National University, Samcheok 25949, Gangwon-do, Republic of Korea

**Keywords:** subjective expectations, cognitive decline, dementia, older adults, longitudinal analysis

## Abstract

This study investigated the impact of subjective expectations of the future (e.g., income, life expectancy, and national policies) on the onset of dementia and mild cognitive impairment by sex and age in middle-aged and older adults. The Korean Longitudinal Study of Aging (KLoSA) data from 2008 to 2020, comprising 4116 people above 45 years, were used. A time-series analysis and multiple panel logistic regression were conducted to highlight subjective expectation trends and their effect on dementia and mild cognitive impairment, respectively. Low subjective expectations of the future negatively affected cognitive impairment (total: odds ratio [OR] = 1.02, 95% confidence interval [CI] = 1.01–1.03) and dementia (total: OR = 1.05, 95% CI = 1.03–1.06), and those of national policies were the biggest risk factors for cognitive impairment (total: OR = 1.17, 95% CI = 1.12–1.22) and dementia (total: OR = 1.10, 95% CI = 1.07–1.13). Individuals about to retire and with low expectations of workability were more likely to develop cognitive impairment (total: OR = 1.03, 95% CI = 1.02–1.06). Subjective expectations of economic downturn also caused cognitive impairment, especially in women (OR = 1.04, 95% CI = 1.01–1.07) and early stage older adults (OR = 1.06, 95% CI = 1.02–1.10). Policymakers must consider the impact of changes in national policies and living environments on cognitive impairment and dementia in older adults.

## 1. Introduction

According to the World Alzheimer’s Report, 2019 [1], there are approximately 50 million people with dementia worldwide and this number is expected to triple to 150 million by 2050 [1]. In particular, the rate of increase in the prevalence of dementia is considerably higher in Korea than in Organization for Economic Co-operation and Development (OECD) countries. By 2050, the average prevalence of dementia in OECD countries is expected to increase to 29.1, while in Korea, to 38.9 [2]. Genetic, medical, and lifestyle factors have long been considered as determinants of dementia. However, in addition to diagnosis, treatment, and rehabilitation, the impact of the relationship between the social structure and social status of organizations and individuals should be considered regarding dementia [3]. According to studies that have analyzed the relationship between social factors and dementia, socioeconomic characteristics, including the level and quality of education, occupation, and income, as well as the residential area and community environment constituting social networks, have been identified as factors related to dementia.

From a life course perspective, anxiety and depressive symptoms appear in middle and old age, because of the individuals’ fear regarding their future. Negative expectations of the future can be associated with lower levels of happiness or despair [4]. Hopelessness regarding the future is a major cause of development of cognitive symptoms of depression [5], while subjective expectations of the future are related to health status [6,7,8]. Moreover, negative expectations regarding the future may lead to substance abuse, alcohol intake, avoidance of life, low persistence, and poor health [9,10]. According to a systematic literature review, those who report anxiety symptoms have a higher risk of developing dementia [11,12,13]. Older adults with depression or anxiety may rapidly develop dementia symptoms [14]. In general, emotional disorders such as anxiety and depression are long-term risk factors for dementia. Additionally, subjective life expectancy is an indicator of future subjective expectations. Subjective life expectancy is the evaluation of an individual’s expectations of their lifetime [15], reflecting their optimistic or pessimistic view toward life [16]. From this perspective, subjective life expectancy affects one’s health through their emotions and moods [8,15]. Moreover, subjective views of economic situation, health status, and well-being as factors of subjective expectations of the future are related to mortality [17,18].

Personal factors, thoughts, and perceptions of various dimensions of the social environment are expressed through one’s expectations of the future [19]. The state is responsible for ensuring the right to health of its citizens; people expect support and protection from the government [20,21]. A country’s policies and systems shape the social, economic, and environmental conditions that influence people’s health and well-being. By prioritizing policies that promote equity, access to healthcare, social welfare, real estate market, and environmental sustainability, as well as preventing economic recession, governments can improve population health outcomes and enhance quality of life.

Nevertheless, a few longitudinal studies have examined measures to prevent cognitive decline and dementia from a sociopsychological and policy perspective. Dementia is chronic and degenerative and is affected more so by social characteristics experienced throughout life than by those of the present time. Therefore, a longitudinal approach is required to identify the causes of dementia [22]. Age is the most specific risk factor for dementia onset. According to a previous study, middle-aged people in their 40s and 60s had a higher fear of dementia than of other serious diseases, such as cancer and cardiovascular disease; however, their confidence in overcoming the disease, expectations of the possibility of maintaining daily life activities, obtaining help from family and neighbors, and medical and economic support were significantly low [23]. Compared to middle-aged adults, older adults are more likely to be affected by their health status because of negative expectations of the future [24]. Therefore, anxiety and depressive symptoms in adulthood and old age are associated with an increased risk for dementia [25]. Older adults in Korea may have greater expectations and concerns regarding the future, from a national perspective, as they experienced aggression and separation from their families during the War of 1950 [24]. Therefore, to maintain the health of older adults and to deal with the rapidly increasing problem of dementia, it is necessary to consider how to cope with life in old age, from a life course perspective.

Studies conducted thus far have mainly focused on the post-onset management of dementia; however, preventive management of dementia is crucial. Although some studies have stated that sociopsychological factors such as depression and anxiety cause dementia, they did not analyze the specific pathways underlying depression and anxiety. Indeed, the more people that practice dementia prevention, the higher their confidence in overcoming the disease, the greater the possibility of maintaining daily life functions, the higher their expectations of obtaining help from family and neighbors, as well as national medical and economic support, and the lower the economic burden of dementia treatment costs [23,26]. In addition, interventions regarding health-promoting behaviors of middle-aged women can induce successful aging awareness and reduce aging anxiety, as well as the fear of dementia [26]. Recently, some studies have been conducted in Korea to analyze the effect of anticipation on dementia from a preventive perspective; however, they had limited generalizability and representability of the sample, because the surveys were conducted only for a specific group [23,26].

Thus, this study attempted to determine whether (a) the change in subjective expectations of the future affects the onset of dementia in middle-aged and older adults, using 12-year representative long-term data and (b) subjective expectations of any factors affect their cognitive function or dementia from the perspective of life course by sex and age. For this, the following hypotheses were proposed: (1) lower subjective expectations of the future negatively impact cognitive impairment or dementia; (2) the relationship between subjective expectations of the future and cognitive impairment or dementia differs based on sex and age; and (3) subjective expectations of the future vary according to the severity of the cognitive impairment (mild cognitive impairment or dementia).

## 2. Materials and Methods

### 2.1. Study Sample and Design

**Figure 1:**
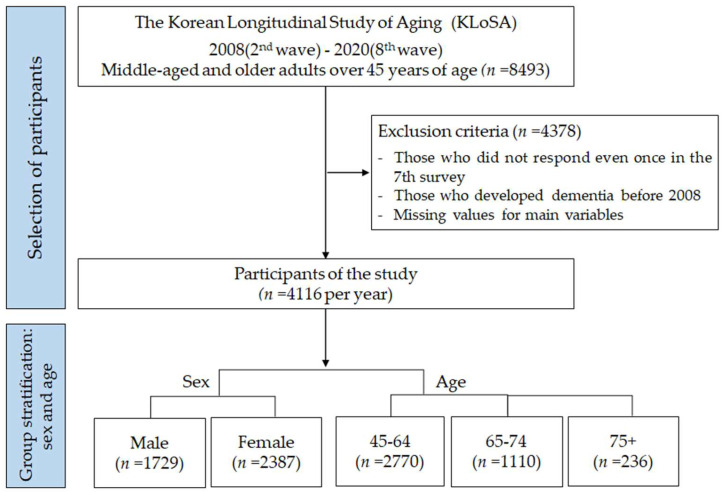
Flow chart of sample selection.

The Korean Longitudinal Study of Aging (KLoSA), conducted by the Korea Labor Institute since 2006, is a nationally representative longitudinal survey of individuals aged 45 years and older. The KLoSA has accumulated basic data through various interdisciplinary studies, measuring and identifying biological phenomena (physical and mental health), in addition to population, social, economic, and psychological phenomena in middle-aged and older adults for effective policy establishment and academic research. This study used the second- to eighth-wave data of the KLoSA, including 4116 people (28,812 cases) over 45 years old who answered related questions every year between 2008 and 2020 (Figure 1). The minimum sample size obtained using the G*POWER 3.1.9.7 (33) program was 992.

### 2.2. Variables

#### 2.2.1. Dependent Variables: Dementia and Mild Cognitive Impairment

The Korean version of the Mini-Mental State Examination (K-MMSE) [27] was used to measure dementia and mild cognitive impairment. The K-MMSE [28,29] assesses general cognitive functioning with scores for orientation, attention and concentration, memory, language, and visuo-constructional functioning. The maximum possible score is 30. In general, a score of 18–23 is associated with mild cognitive impairment and that of <18 is associated with dementia [27,29]. In this study, the scores were coded as follows: <18 = 1 (dementia); 18–23 = 1 (mild cognitive impairment), and >24 = 0.

#### 2.2.2. Independent Variable: Subjective Expectation

Subjective expectations regarding the future were assessed using 13 items from the database. These items measure a continuum of subjective probabilities by obtaining responses to the following components.

(1) Legacy gift (when I think about all the assets that I have, I can leave more than about USD 90000). (2) Legacy gift (I can receive more than about USD 90000). (3) Work expectancy (what is the probability that you will work to be [55 (if age is 45–59)/60 (if age is 50–54)/65 (if age is 55–59)/next five years (if age is 65 or more)]? The target age was determined by respondents’ current age. (4) Life expectancy (what is the probability that you will live five years longer than your current age). (5) The standard of living in the future (I am going to lower my standard of living in the future). (6) Socio-economic level of their children (I think that the younger generation can live in a better economic/social environment than our generation). (7) Security in old age (I can guarantee my old age in the country). (8) National pension (the national pension is helpful in my old age). (9) Basic pension (the basic pension is helpful in my old age). (10) National health insurance. (11) National long-term care insurance. (12) Recession (in the next 10 years, Korea’s economy is likely to suffer from a severe recession). (13) Stable real estate market (in the next 10 years). All 13 questions were answered using a continuous 10-point scale (0–10).

These 13 items were classified into 7 subdomains of subjective expectations, as follows: (i) income (1, 2); (ii) work (3); (iii) life expectancy (4); (iv) standard of living (5, 6, 7); (v) national policies (8, 9, 10, 11); (vi) recession (12); and (vii) real estate markets (13).

#### 2.2.3. Other Variables

The covariates comprised the demographics, health status, and health behavior factors. Demographic characteristics included sex (1: male, 2: female), age group (1: 45–64, 2: 65–74, 3: >75 years), marital status (1: married, 2: divorced/widowed/separated/single), monthly income (1: high, 2: middle–high, 3: middle–low, 4: low), level of education (1: college or above, 2: high school, 3: under middle school), and type of health insurance (1: national health insurance, 2: medical aid). Health status characteristics included self-rated health status (1: good, 2: moderate, 3: poor), number of chronic diseases (0, 1, 2, >2), and health behavior factors, including drinking (1: no, 2: yes) and smoking (1: no, 2: yes).

### 2.3. Statistical Analysis

First, we conducted a time-series analysis to examine the frequencies of the seven domains of subjective expectations by sex and age. Second, we calculated descriptive statistics on demographics, health status, and health behavior factors in 2008 and 2020. Third, we used the chi-square test to confirm the differences in dementia and mild cognitive impairment based on covariates. Additionally, we used a multiple panel logistic regression analysis to analyze the effects of the subjective expectations of dementia and mild cognitive impairment and applied a fixed-effects model based on the Hausman test results. For convenience, the analysis was conducted through reverse coding of the subjective expectation and seven subdomains. The covariates were included in the analysis. Statistical analyses were performed using STATA version 17.0. For all analyses, the statistical significance was *p* ≤ 0.05, two-tailed.

## 3. Results

### 3.1. Trend of the Seven Domains of Subjective Expectation

**Figure 2:**
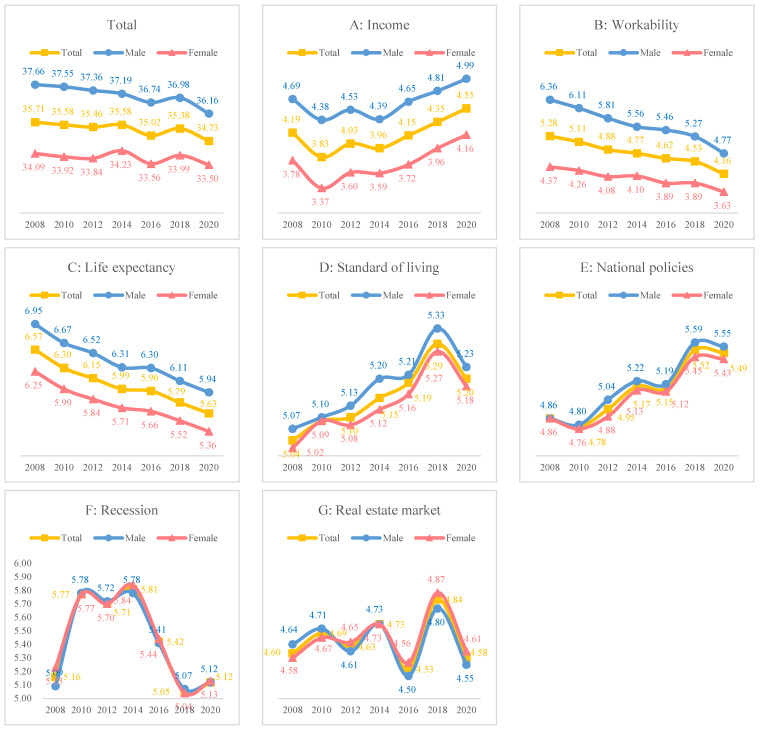
Trend of the seven domains of subjective expectation of the future by sex from 2008 to 2020.

Figure 2 presents the trends in the seven domains of subjective expectations of the future by sex. Subjective expectancy decreased and men had higher subjective expectancy scores than women. As for the seven subdomains, expectations of income (A), standard of living (D), and national policies (E) increased, while those for work (B) and life expectancy (C) decreased. Expectations of a recession (F) and the real estate market (G) fluctuated.

**Figure 3:**
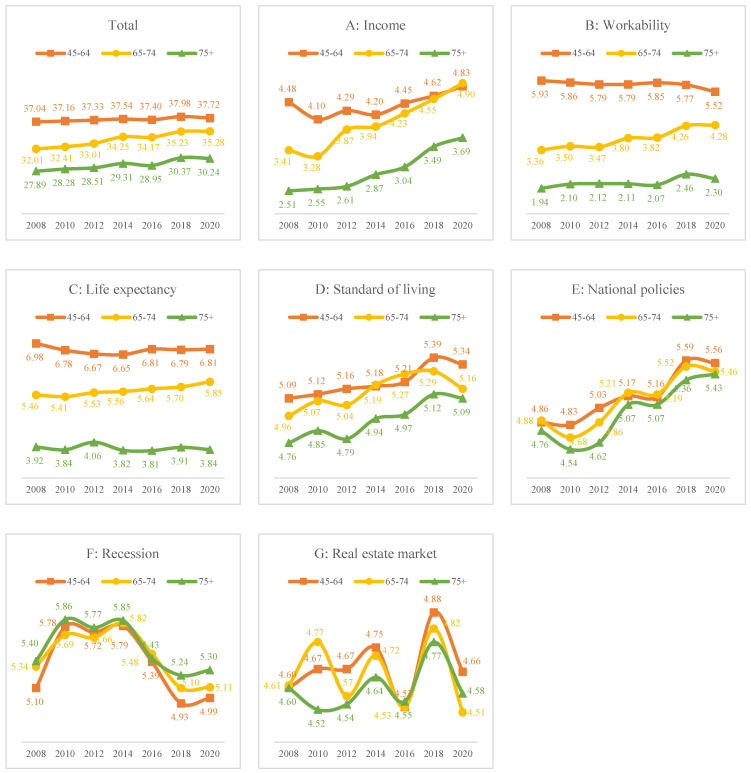
Trend of the seven domains of subjective expectation of the future by age group from 2008 to 2020.

Figure 3 depicts the trends in the seven domains of subjective expectations by age group. The 45–64 years age group showed the highest level of subjective expectancy in the seven subdomains, followed by the 65–74 years age group and the 75 years or over group. From 2008 to 2020, the subjective expectation of the 45–64 year age group was stable, but that of the 65–74 and 75 or over age groups increased. As for the seven subdomains, expectations of income (A), standard of living (D), and national policies (E) increased, whereas expectations of work and life expectancy (C) remained stable. The expectation of a recession (F) increased until 2014, followed by a decline. Expectations of the real estate market (G) repeatedly increased and decreased.

### 3.2. General Characteristics

Table 1 presents the general characteristics of the sample in 2008. Regarding sex, 46.2% were men and 53.8% were women. The proportion of older adults aged 75 or older was 3.6%; 86.6% were married; and 13.4% were divorced/widowed. As for monthly income, 16.3% were in the low-income group. Medical aid (MA) beneficiaries accounted for 3.8%. The proportions of people with drinking and smoking habits were 43.3% and 20.3%, respectively. Good health status was reported by 44.6% and chronic health status by 54.1%.

### 3.3. Differences in Dementia and Mild Cognitive Impairment

Table 2 shows the differences in dementia and mild cognitive impairment according to general characteristics. Compared with 2008, the rates of dementia and mild cognitive impairment increased in 2020. Women, those who were in the age group of 75 years or older, those who were divorced/widowed, those with low income, those with low education levels, MA beneficiaries, and those with poor health status and chronic diseases reported higher rates of dementia and mild cognitive impairment. Trends in dementia and mild cognitive impairment are presented in Appendix A and Appendix B.

### 3.4. Effects of Subjective Expectation on Dementia and Mild Cognitive Impairment

Table 3 presents the panel regression analysis results of the effects of subjective expectations on dementia and mild cognitive impairment by sex and age. Subjective expectation showed a significant effect on dementia and mild cognitive impairment both in men (dementia: odds ratio [OR] = 1.03, 95% confidence interval [CI] = 1.01–1.05; mild cognitive impairment: OR = 1.03, 95% CI = 1.02–1.05) and women (dementia: OR = 1.05, 95% CI = 1.04–1.06; mild cognitive impairment: OR = 1.03, 95% CI = 1.02–1.04). In men, life expectancy (OR = 1.10, 95% CI = 1.04–1.19) and expectation of national policies (OR = 1.18, 95% CI = 1.08–1.29) significantly impacted dementia. Expectation of income (OR = 1.03, 95% CI = 1.01–1.05), workability (OR = 1.05, 95% CI = 1.01–1.09), life expectancy (OR = 1.07, 95% CI = 1.03–1.11), and national policy (OR = 1.12, 95% CI = 1.06–1.18) significantly impacted mild cognitive impairment. In women, the lower the expectation of income (OR = 1.07, 95% CI = 1.03–1.09), life expectancy (OR = 1.07, 95% CI = 1.03–1.11), and national policies (OR = 1.18, 95% CI = 1.11–1.24), the higher the risk of dementia and the lower the expectation of income (OR = 1.04, 95% CI = 1.01–1.15), workability (OR = 1.05, 95% CI = 1.02–1.08), life expectancy (OR = 1.05, 95% CI = 1.02–1.08), national policy (OR = 1.09, 95% CI = 1.06–1.13), and recession (OR = 1.04, 95% CI = 1.01–1.07), the higher the risk of mild cognitive impairment.

Subjective expectations significantly affected dementia and mild cognitive impairment in all age groups. In the 45–64 years age group, life expectancy (OR = 1.15, 95% CI = 1.05–1.26) and expectation of national policies (OR = 1.16, 95% CI = 1.02–1.32) showed a significant effect on dementia. The expectation of workability (OR = 1.08, 95% CI = 1.04–1.12), life expectancy (OR = 1.06, 95% CI = 1.01–1.10), and national policy (OR = 1.13, 95% CI = 1.07–1.19) significantly influenced mild cognitive impairment. In the 65–74 years age group, life expectancy (OR = 1.09, 95% CI = 1.02–1.16), standard of living (OR = 0.91, 95% CI = 0.83–0.99), and national policies (OR = 1.18, 95% CI = 1.10–1.28) significantly affected dementia; income expectancy (OR = 1.05, 95% CI = 1.03–1.07), life expectancy (OR = 1.07, 95% CI = 1.04–1.10), national policies (OR = 1.08, 95% CI = 1.04–1.13), and recession (OR = 1.06, 95% CI = 1.02–1.10) significantly impacted mild cognitive impairment. Finally, in the group of 75 years or over, income (dementia: OR = 1.09, 95% CI = 1.05–1.13; mild cognitive impairment: OR = 1.06, 95% CI = 1.03–1.09), life expectancy (dementia: OR = 1.06, 95% CI = 1.01–1.10; mild cognitive impairment: OR = 1.03, 95% CI = 1.01–1.07), and national policies (dementia: OR = 1.15, 95% CI = 1.08–1.23; mild cognitive impairment: OR = 1.08, 95% CI = 1.03–1.14) significantly affected both dementia and mild cognitive impairment, while real estate market (OR = 0.93, 95% CI = 0.88–0.99) significantly impacted dementia.

## 4. Discussion

In this study, the effects of subjective expectations of the future, regarding living standards, workability, life expectancy, government policies, economic recession, and real estate market, on cognitive decline and dementia were comprehensively evaluated from a sociological perspective. The relationship between seven areas of subjective expectations and the development of cognitive impairment and dementia in middle-aged and older adults was investigated using KLoSA data. In addition, the study contributes to dementia prevention by highlighting the aspects of subjective expectations with the greatest influence on the risk of cognitive dysfunction and dementia, according to sex and life stage.

From 2008 to 2020, overall subjective expectations of the future slightly decreased. Women and older adults had lower subjective expectations. Expectations of living standards, personal wealth, and government policies increased, whereas those of workability and life expectancy tended to decrease. Workability and life expectancy anxiety among older adults remains an issue to be addressed in the future. Expectations of living standards have steadily improved since 2008, but have declined since 2018. The expectations of economic downturns and real estate policies exhibited irregular patterns. This may be attributed to global economic slowdown and trade tensions. In particular, the real estate market in Korea appears to be unstable due to frequent changes in government policies, in response to real estate market downturns. 

According to the results of this study, low subjective expectations of the future in middle and old age negatively affect cognitive impairment and dementia. Specifically, in all age groups over 45 years, lower expectations regarding life expectancy and government policies induced both cognitive impairment and dementia. A previous study reported that low life expectancy increases the risk of mortality [15]; this study explains the mechanism between the two. Previous studies have demonstrated higher mortality rates among individuals with cognitive impairment or dementia [30]. In particular, the mortality rate among older adults with dementia is high in extremely disturbing situations, such as the COVID-19 pandemic [31]. The results of this study showed that, a low life expectancy was associated with a high risk of cognitive decline and dementia. Therefore, this study highlighted the mechanisms that could mediate cognitive decline and dementia in the relationship between low life expectancy and mortality. It is necessary to reduce the risk factors for low life expectancy, in order to lower the incidence of cognitive impairment and dementia and to reduce the risk of mortality. As factors affecting subjective life expectancy, various demographic characteristics, including age, sex, socioeconomic position, educational level [32,33], health status [34], health promotion [35], and social relationships [36], can be addressed through policies.

Subjective expectations of government policies were the biggest risk factors for cognitive impairment and dementia. This indicates that the stability of social security and the state’s medical insurance system can contribute to the prevention of cognitive decline and dementia. For example, high-deductible health plans (HDHPs) in the United States reduced access to treatment and rehabilitation, exacerbating cognitive impairment [37]. Studies have also reported that adults with multiple chronic diseases in late middle age, nearing Medicare enrollment, and people of all races without private insurance are more likely to experience depression and anxiety symptoms [38,39]. In China, income from the National Pension in old age is highly related to cognitive function [40]. Therefore, strengthening health insurance and social security can prevent cognitive decline and dementia in older adults.

Some subjective expectation items differed based on age group. In individuals who are about to retire before the age of 65 years, low expectations of workability were more likely to cause cognitive impairment. In Korea, as of 2021, through the recent government policy to create jobs for older adults, the retirement age is 72.3 years, 7.8 years higher than the OECD average [41]. However, the problem of declining job quality is still highlighted, whereby the older the age, the lower the income level and the more unstable the employment patterns [42]. Therefore, by reflecting on the results of this study, the risk of cognitive impairment among older adults can be reduced by implementing countermeasures regarding jobs for this population, as well as individuals’ working capacity before old age.

Subjective expectations of an economic downturn can also cause cognitive impairment, especially in women and older adults in early stages. In this regard, anxiety about personal assets mostly affected cognitive impairment after the age of 65, when it was difficult to work, which led to an increased risk of dementia in adults aged 75 and over. In other words, an individual’s socioeconomic status and current economic recession level, such as wealth and income levels, are important factors influencing cognitive impairment and dementia. A previous study found that the low income of female nurses working after the official retirement age predicted cognitive decline at present and up to two years later [43]. Older adults with high income at an old age may have more practical opportunities to improve their cognitive function, because they have more favorable psychological, economic, and geographical access to counseling or medical services to improve their impaired cognitive function.

In 2018, the Korean government announced community care and expanded and reorganized long-term care services, showing continued interest in caring for older adults with dementia in the community [44]. It mainly supports the improvement of daily life and quality of life [30]. To date, academia has focused on the post-onset management of dementia and there is a growing interest in the approaches to manage symptoms at onset. However, this study emphasizes the need to prevent cognitive impairment and dementia before symptom onset. To reduce the onset of cognitive decline and dementia, support regarding the fear of livelihood, such as work and assets, is required from middle to old age.

Regarding the first hypothesis, all subdomains of subjective expectations, excluding the real estate market, significantly influenced cognitive impairment. Regarding the second hypothesis, sex- and age-based differences were observed in the relationship between subjective expectations and cognitive impairment. Economic downturns have been found to potentially induce cognitive impairment among women. Those under 65 years of age showed a higher likelihood of cognitive impairment, owing to low expectations of workability. Regarding the third hypothesis, low expectations of a recession and workability impacted cognitive impairment.

This study had several limitations. First, according to previous studies, cognitive impairment and dementia in older adults are affected by social relationships and support [40], but the research model described in this study did not include social relationships. Therefore, additional follow-up studies on the effect of interventions on social relationships are needed to elucidate the mechanism underlying the relationship between subjective expectations and dementia. Second, a hierarchical analysis was conducted by sex and age group on the relationship between subjective expectations of the future and cognitive impairment. According to our results, expectations regarding income level affect cognitive impairment in older adults. Therefore, in future research, it is necessary to present specific policy implications by conducting a stratified analysis by income group.

## 5. Conclusions

This study found that raising positive expectations of the future is important for improving health by preventing cognitive impairment and dementia in middle-aged and older adults. Future policymakers should consider that changes in national policies and individual living environments affect the health of older adults.

## Figures and Tables

**Table 1:** General characteristics of the study population at baseline.

	2008	
	n	%
Total	4116	100.0
Sex		
Male	1729	46.2
Female	2387	53.8
Age group		
55–64	2770	76.7
65–75	1110	19.7
75+	236	3.6
Marital status		
Married	3509	86.6
Single/divorced/widowed	607	13.4
Monthly income		
High	1011	28.2
Middle–high	1090	28.8
Middle–low	1181	26.7
Low	834	16.3
Level of education		
College and higher	405	12.3
High school	1262	35.2
Under middle school	2449	52.5
Health insurance		
NHI ^1^	3961	96.2
MA ^2^	155	3.8
Drinking		
No	2480	56.7
Yes	1636	43.3
Smoking		
No	3395	79.7
Yes	721	20.3
Health status		
Good	1650	44.6
Moderate	1577	36.3
Poor	889	19.1
Chronic diseases		
No	2046	54.1
Yes	2070	45.9

^1^ National health insurance, ^2^ Medical aids.

**Table 2:** Differences in dementia and mild cognitive impairment by general characteristics.

	2008							2020						
	Dementia		Mild Cognitive Impairment		Normal		*p*	Dementia		Mild Cognitive Impairment		Normal		*p*
	n	%	n	%	n	%		n	%	n	%	n	%	
Total	136	2.5	581	11.9	3399	85.6		404	7.7	845	18.4	2733	73.9	
Sex														
Male	21	0.8	138	6.3	1570	92.9	<0.001	109	4.3	322	17.0	1239	78.7	<0.001
Female	115	3.9	443	16.5	1829	79.6		295	10.7	523	19.5	1494	69.8	
Age group (years)														
55–64	25	0.8	232	7.5	2513	91.7	<0.001	12	1.1	83	9.5	865	89.4	<0.001
65–75	71	6.2	268	24.8	771	69.0		54	3.4	276	16.7	1150	79.9	
75+	40	17.8	81	35.0	115	47.2		338	22.5	486	32.0	718	45.5	
Marital status														
Married	83	1.8	431	10.2	2995	88.0	<0.001	181	4.1	549	16.2	2233	79.7	<0.001
Single/divorced/widowed	53	7.0	150	22.5	404	70.5		223	19.1	296	25.1	500	55.8	
Monthly income														
High	8	0.5	64	5.2	939	94.3	<0.001	50	3.8	99	10.7	650	85.5	<0.001
Middle–high	20	1.3	101	8.5	969	90.2		44	3.3	156	15.0	753	81.7	
Middle–low	32	2.2	195	14.5	954	83.3		79	6.6	205	19.1	706	74.3	
Low	76	8.5	221	25.0	537	66.5		229	17.6	383	29.5	617	52.9	
Level of education														
College and higher	0	0.0	8	1.7	397	98.3	<0.001	8	1.8	41	9.7	352	88.5	<0.001
High school	4	0.2	61	4.5	1197	95.3		32	2.0	136	9.8	1052	88.2	
Under middle school	132	4.6	512	19.2	1805	76.2		364	13.5	668	26.9	1329	59.6	
Health insurance														
NHI ^1^	120	2.3	541	11.4	3300	86.3	<0.001	366	7.2	790	17.8	2652	75.0	<0.001
MA ^2^	16	8.5	40	23.6	99	67.8		38	18.8	55	29.1	81	52.1	
Drinking														
No	109	3.4	442	15.7	1929	80.8	<0.001	374	10.7	662	20.3	1805	69.0	<0.001
Yes	27	1.2	139	6.8	1470	91.9		30	1.7	183	14.5	928	83.8	
Smoking														
No	128	2.9	519	13.1	2748	84.0	<0.001	385	8.0	810	19.3	2501	72.7	<0.001
Yes	8	0.9	62	6.9	651	92.2		19	5.1	35	9.5	232	85.4	
Health status														
Good	11	0.5	71	3.5	1568	96.0	<0.001	25	1.8	97	8.6	844	89.6	<0.001
Moderate	55	2.8	263	14.8	1259	82.4		104	4.1	407	18.7	1427	77.3	
Poor	70	6.7	247	25.6	572	67.7		275	23.3	341	29.4	462	47.4	
Chronic diseases														
No	27	1.0	189	7.5	1830	91.5	<0.001	34	2.1	131	10.4	860	87.5	<0.001
Yes	109	4.3	392	17.0	1569	78.7		370	10.0	714	21.6	1873	68.4	

^1^ National health insurance, ^2^ Medical aids.

**Table 3:** Panel regression analysis results of the effect of subjective expectation on dementia and mild cognitive impairment by sex and age.

Sex	Total				Men				Women			
	Dementia		Mild Cognitive Impairment		Dementia		Mild Cognitive Impairment		Dementia		Mild Cognitive Impairment	
	OR	95% CI	OR	95% CI	OR	95% CI	OR	95% CI	OR	95% CI	OR	95% CI
Total	1.05	1.03–1.06	1.02	1.01–1.03	1.03	1.01–1.05	1.03	1.02–1.05	1.05	1.04–1.06	1.03	1.02–1.04
Income	1.05	1.02–1.07	1.03	1.02–1.05	0.99	0.95–1.04	1.03	1.01–1.05	1.07	1.03–1.09	1.04	1.01–1.05
Workability	1.00	0.97–1.04	1.03	1.02–1.06	0.99	0.93–1.07	1.05	1.01–1.09	1.01	0.97–1.05	1.05	1.02–1.08
Life expectancy	1.08	1.05–1.12	1.06	1.04–1.08	1.10	1.04–1.19	1.07	1.03–1.11	1.07	1.03–1.11	1.05	1.02–1.08
Standard of living	1.01	0.96–1.06	0.97	0.94–1.01	0.99	0.89–1.09	0.97	0.91–1.03	1.01	0.96–1.08	0.98	0.94–1.02
National policies	1.17	1.12–1.22	1.10	1.07–1.13	1.18	1.08–1.29	1.12	1.06–1.18	1.18	1.11–1.24	1.09	1.06–1.13
Recession	0.99	0.95–1.03	1.04	1.01–1.06	1.01	0.94–1.07	1.03	0.99–1.07	0.99	0.95–1.04	1.04	1.01–1.07
Real estate market	0.98	0.94–1.02	0.99	0.97–1.01	0.96	0.89–1.04	0.96	0.92–1.00	0.98	0.93–1.03	0.99	0.96–1.02
**Age**	**45–64**				**65–74**				**75+**			
	**Dementia**		**Mild** **cognitive** **impairment**		**Dementia**		**Mild** **cognitive** **impairment**		**Dementia**		**Mild** **cognitive** **impairment**	
	**OR**	**95% CI**	**OR**	**95% CI**	**OR**	**95% CI**	**OR**	**95% CI**	**OR**	**95% CI**	**OR**	**95% CI**
Total	1.05	1.02–1.09	1.04	1.02–1.05	1.04	1.02–1.06	1.03	1.02–1.04	1.05	1.03–1.06	1.03	1.02–1.04
Income	0.96	0.91–1.02	1.01	0.99–1.04	1.03	0.98–1.07	1.05	1.03–1.07	1.09	1.05–1.13	1.06	1.03–1.09
Workability	0.98	0.91–1.06	1.08	1.04–1.12	1.02	0.96–1.08	1.01	0.98–1.04	1.00	0.95–1.06	1.00	0.96–1.03
Life expectancy	1.15	1.05–1.26	1.06	1.01–1.10	1.09	1.02–1.16	1.07	1.04–1.10	1.06	1.01–1.10	1.03	1.01–1.07
Standard of living	1.09	0.94–1.27	0.96	0.90–1.03	0.91	0.83–0.99	0.96	0.91–1.01	1.02	0.96–1.09	1.00	0.95–1.06
National policies	1.16	1.02–1.32	1.13	1.07–1.19	1.18	1.10–1.28	1.08	1.04–1.13	1.15	1.08–1.23	1.08	1.03–1.14
Recession	0.97	0.88–1.08	1.02	0.99–1.07	1.00	0.94–1.07	1.06	1.02–1.10	0.98	0.93–1.03	1.00	0.96–1.05
Real estate market	1.06	0.94–1.18	0.98	0.93–1.02	0.99	0.93–1.07	0.99	0.95–1.02	0.93	0.88–0.99	0.98	0.93–1.02

## Data Availability

The dataset generated and/or analyzed during the current study is publicly available and is available in the ‘Korean Longitudinal Study of Aging (KLoSA)’ for the longitudinal study of aging by the Korean Employment Information Service from https://survey.keis.or.kr/klosa/klosa04.jsp (accessed on 13 May 2024).

## Appendix A. Trend of Dementia from 2008 to 2020

	**Dementia**													
	**2008**		**2010**		**2012**		**2014**		**2016**		**2018**		**2020**	
	**n**	**%**	**n**	**%**	**n**	**%**	**n**	**%**	**n**	**%**	**n**	**%**	**n**	**%**
Total	136	2.5	180	3.2	178	3.2	244	4.4	273	5.0	368	6.6	404	7.7
Sex														
Male	21	0.8	31	1.3	28	1.3	49	2.0	62	2.5	109	4.5	109	4.3
Female	115	3.9	149	4.8	150	4.9	195	6.4	211	7.1	259	8.4	295	10.7
Age group (years)														
55–64	25	0.8	24	0.8	25	0.9	19	0.9	13	0.8	28	1.7	12	1.1
65–75	71	6.2	85	6.7	71	5.1	72	4.9	66	4.5	62	3.9	54	3.4
75+	40	17.8	71	18.8	82	14.8	153	19.5	194	19.5	278	20.7	338	22.5
Marital status														
Married	83	1.8	107	2.2	112	2.3	121	2.5	138	3.1	187	3.9	181	4.1
Single/ divorced/widowed	53	7.0	73	8.5	66	7.6	123	12.8	235	12.6	181	16.2	223	19.1
Monthly income														
High	8	0.5	18	0.9	18	1.1	17	1.1	23	1.8	51	3.3	50	3.8
Middle–high	20	1.3	20	1.8	24	1.8	26	2.1	27	2.2	40	3.0	44	3.3
Middle–low	32	2.2	41	2.7	37	2.6	56	3.4	68	4.2	95	5.8	79	6.6
Low	76	8.5	101	10.5	99	9.4	145	13.1	155	14.3	182	16.7	229	17.6
Level of education														
College and higher	0	0.0	1	0.5	2	0.8	3	0.9	3	1.5	10	2.1	8	1.8
High school	4	0.2	7	0.5	11	0.6	15	0.8	23	1.2	33	2.0	32	2.0
Under middle school	132	4.6	172	5.7	165	5.6	226	7.8	247	8.7	325	11.2	364	13.5
Health insurance														
NHI ^1^	120	2.3	157	2.9	157	2.9	209	3.8	244	4.6	331	6.0	366	7.2
MA ^2^	16	8.5	23	0.9	21	9.8	35	15.1	29	13.5	37	22.1	38	18.8
Drinking														
No	109	3.4	158	4.7	150	4.5	207	6.1	241	7.1	320	9.0	374	10.7
Yes	27	1.2	22	1.0	28	1.4	37	1.8	32	1.6	48	2.5	30	1.7
Smoking														
No	128	2.9	162	3.5	164	3.6	224	4.6	254	5.3	348	6.9	385	8.0
Yes	8	0.9	180	1.8	14	1.7	20	3.3	19	2.7	20	4.2	19	5.1
Health status														
Good	11	0.5	22	1.1	15	0.6	19	1.1	16	0.9	28	1.7	25	1.8
Moderate	55	2.8	52	2.3	57	2.5	60	2.3	72	2.7	115	4.1	104	4.1
Poor	70	6.7	106	9.5	106	10.3	165	14.7	185	16.8	225	18.7	275	23.3
Chronic diseases														
No	27	1.0	30	1.2	28	1.2	32	1.6	34	2.0	44	2.7	34	2.1
Yes	109	4.3	150	4.9	150	4.8	212	6.1	239	6.6	324	8.5	370	10.0
^1^ National Health Insurance, ^2^ Medical aids.														

## Appendix B. Trend of Mild Cognitive Impairment from 2008 to 2020

	**Mild Cognitive Impairment**													
	**2008**		**2010**		**2012**		**2014**		**2016**		**2018**		**2020**	
	**n**	**%**	**n**	**%**	**n**	**%**	**n**	**%**	**n**	**%**	**n**	**%**	**n**	**%**
Total	581	11.9	584	12.2	575	11.0	692	13.8	722	14.2	816	16.8	845	18.4
Sex														
Male	138	6.3	158	7.3	167	6.8	224	9.8	218	9.4	277	13.1	322	17.0
Female	443	16.5	426	16.3	408	14.6	468	17.2	504	18.3	539	19.9	523	19.5
Age group (years)														
55–64	232	7.5	197	7.4	147	5.4	163	7.5	114	6.3	113	8.1	83	9.5
65–75	268	24.8	278	23.4	233	17.3	277	19.0	277	19.0	284	18.2	276	16.7
75+	81	35.0	109	29.0	195	34.5	252	32.0	331	31.1	419	31.9	486	32.0
Marital status														
Married	431	10.2	423	10.4	399	9.2	484	11.8	485	11.8	556	14.8	549	16.2
Single/divorced/widowed	150	22.5	161	22.2	176	20.2	208	22.7	237	23.7	260	23.6	296	25.1
Monthly income														
High	64	5.2	81	6.2	56	3.5	88	7.8	86	7.6	81	7.4	99	10.7
Middle–high	101	8.5	91	8.7	102	8.1	110	9.7	105	9.3	139	13.1	156	15.0
Middle–low	195	14.5	175	13.2	158	13.0	200	13.9	220	15.5	252	19.3	205	19.1
Low	221	25.0	237	28.1	259	25.0	294	27.2	311	27.8	344	30.8	383	29.5
Level of education														
College and higher	8	1.7	20	4.4	10	2.1	17	3.4	21	3.7	27	6.9	41	9.7
High school	61	4.5	86	5.9	72	4.5	106	7.4	96	6.3	137	9.6	136	9.8
Under middle school	512	19.2	478	18.5	493	17.9	569	21.1	605	22.7	652	24.7	668	26.9
Health insurance														
NHI ^1^	541	11.4	525	11.0	524	10.4	647	13.5	667	13.6	759	16.2	790	17.8
MA ^2^	40	23.6	59	37.6	51	23.5	45	20.7	55	25.9	57	30.3	55	29.1
Drinking														
No	442	15.7	423	14.8	421	13.3	508	16.2	543	16.8	623	19.0	662	20.3
Yes	139	6.8	161	8.6	154	7.7	184	10.2	179	10.1	193	13.0	183	14.5
Smoking														
No	519	13.1	518	13.4	513	11.8	629	14.8	678	15.3	757	17.3	810	19.3
Yes	62	6.9	66	7.4	62	7.4	63	8.8	44	6.5	59	12.8	35	9.5
Health status														
Good	71	3.5	105	6.3	78	4.3	100	6.4	92	5.8	113	8.7	97	8.6
Moderate	263	14.8	228	12.2	241	11.1	303	13.5	319	13.6	372	16.5	407	18.7
Poor	247	25.6	251	25.9	256	24.5	289	27.3	311	28.7	331	28.6	341	29.4
Chronic diseases														
No	189	7.5	176	8.2	145	6.8	162	8.6	145	8.2	144	10.6	131	10.4
Yes	392	17.0	408	15.9	430	14.2	530	17.1	577	17.4	672	19.6	714	21.6
^1^ National health insurance, ^2^ Medical aids.														
